# Microvolt T-Wave Alternans Is Modulated by Acute Low-Level Tragus Stimulation in Patients With Ischemic Cardiomyopathy and Heart Failure

**DOI:** 10.3389/fphys.2021.707724

**Published:** 2021-07-23

**Authors:** Kanchan Kulkarni, Stavros Stavrakis, Khaled Elkholey, Jagmeet P. Singh, Kimberly A. Parks, Antonis A. Armoundas

**Affiliations:** ^1^Cardiovascular Research Center, Massachusetts General Hospital, Boston, MA, United States; ^2^Heart Rhythm Institute, The University of Oklahoma Health Sciences Center, Oklahoma City, OK, United States; ^3^Cardiology Division, Cardiac Arrhythmia Service, Massachusetts General Hospital, Boston, MA, United States; ^4^Cardiology Division, Brigham and Women’s Hospital, Boston, MA, United States; ^5^Institute for Medical Engineering and Science, Massachusetts Institute of Technology, Cambridge, MA, United States

**Keywords:** heart failure, ventricular arrhythmias, vagal stimulation, T-wave alternans, spectral method

## Abstract

**Aims:** Microvolt T-wave alternans (TWA), an oscillation in T-wave morphology of the electrocardiogram (ECG), has been associated with increased susceptibility to ventricular tachy-arrhythmias, while vagus nerve stimulation has shown promising anti-arrhythmic effects in *in vivo* and *ex vivo* animal studies. We aimed to examine the effect of non-invasive, acute low-level tragus stimulation (LLTS) on TWA in patients with ischemic cardiomyopathy and heart failure.

**Methods:** 26 patients with ischemic cardiomyopathy (left ventricular ejection fraction <35%) and chronic stable heart failure, previously implanted with an automatic implantable cardioverter defibrillator (ICD) device with an atrial lead (dual chamber ICD or cardiac resynchronization therapy defibrillator), were enrolled in the study. Each patient sequentially received, (1) Sham LLTS (electrode on tragus, but no stimulation delivered) for 5 min; (2) Active LLTS at two different frequencies (5 and 20 Hz, 15 min each); and (3) Active LLTS, during concomitant atrial pacing at 100 bpm at two different frequencies (5 and 20 Hz, 15 min each). LLTS was delivered through a transcutaneous electrical nerve stimulation device (pulse width 200 μs, frequency 5/20 Hz, amplitude 1 mA lower than the discomfort threshold). TWA burden was assessed using continuous ECG monitoring during sham and active LLTS in sinus rhythm, as well as during atrial pacing.

**Results:** Right atrial pacing at 100 bpm led to significantly heightened TWA burden compared to sinus rhythm, with or without LLTS. Acute LLTS at both 5 and 20 Hz, during sinus rhythm led to a significant rise in TWA burden in the precordial leads (*p* < 0.05).

**Conclusion:** Acute LLTS results in a heart-rate dependent increase in TWA burden.

## Highlights

- Low level tragus stimulation (LLTS) significantly modulates microvolt T-wave alternans (TWA) in patients with ischemic cardiomyopathy and heart failure.
- Acute LLTS results in a heart rate (HR) dependent increase in TWA burden, wherein increased HR leads to an increase in TWA burden.
- The distinct and significant modulation of TWA by acute LLTS, in conjunction with the possible bimodal effect of LLTS, provides insights for a more comprehensive trial to evaluate the effects of chronic LLTS in patients susceptible to ventricular tachy-arrhythmias, and investigate the potential of patient-specific stimulation strategies in evoking the optimal response to LLTS, in a population at high risk for sudden cardiac death.

## Introduction

Heightened sympathetic activity has been associated with the generation of ventricular tachy-arrhythmias. Although the effect of the sympathetic nervous system stimulation on the heart is complex and is governed by the state of the myocardium, interventions that reduce cardiac sympathetic activity have been shown to protect against arrhythmias (Schwartz and Zipes, 2000; Rubart and Zipes, 2005), whereas those that enhance sympathetic activity provoke them (Rubart and Zipes, 2005; Billman, 2006). Consistent with these findings, β-blocker therapy, used for reducing cardiac sympathetic activity by blocking the effects of epinephrine and norepinephrine, has been shown to reduce sudden cardiac death (SCD) in patients with heart failure (HF) (MERIT-HF, 1999; Poole-Wilson et al., 2003).

Sharp upsurges in T-wave alternans (TWA), an oscillation in T-wave morphology of the electrocardiogram (ECG), immediately preceding spontaneous ventricular tachycardia or fibrillation (VT/VF) have been documented in body-surface ECGs in patients with coronary artery disease (Shusterman et al., 2006), as well as patients hospitalized for acute HF (Nearing et al., 2012). Increase in microvolt TWA was shown to correlate with elevated sympathetic activity in humans (Verrier and Antzelevitch, 2004; Lampert et al., 2005), while the amplitude of TWA was diminished with β-blockers (Klingenheben et al., 2001; Rashba et al., 2002; Komiya et al., 2005). In patients with ventricular tachy-arrhythmias who underwent TWA testing, acute administration of β-blockers metoprolol and dl-sotalol reduced overall TWA amplitude by 35% and 38%, respectively (Klingenheben et al., 2001). TWA has also been observed to occur at significantly lower heart rates (HRs), with peak alternans level increasing with sympathetic stimulation compared to baseline, in basic science studies (Ng et al., 2007).

These studies indicate that TWA can be, at least in part, modulated by sympathetic activity. Furthermore, it has been proposed that in addition to sympathetic activation, parasympathetic withdrawal may also contribute to HF (Olshansky et al., 2008). While in Holter monitoring chronic vagal nerve stimulation (VNS) reduced TWA and the incidence of VT (Libbus et al., 2016), it remains unclear whether targeted use of VNS during heightened levels of TWA, could prevent the onset of VT/VF.

We have recently shown that VNS can be delivered non-invasively by stimulating the auricular branch of the vagus nerve at the tragus of the ear (Stavrakis et al., 2015, 2020a,b). Specifically, in our recent proof-of-concept randomized study in humans, we showed that in patients with atrial fibrillation (AF), low-level transcutaneous vagus nerve stimulation (LLTS), delivered at the tragus of the ear, where the auricular branch of the vagus nerve is located, for just one hour, significantly suppressed AF and decreased systemic inflammatory cytokines (Stavrakis et al., 2015), and similar effects were also observed with chronic LLTS for 6 months (Stavrakis et al., 2020b).

Since, the most common cause of death in patients with ischemic cardiomyopathy (ICM) is SCD, here, the objective of this study is to examine the effect of LLTS on TWA, in patients with ICM and HF (New York Heart Association, NYHA, class II), which are known to have the highest risk of SCD (Myerburg and Junttila, 2012). In this patient population, implantable cardioverter defibrillators reduce mortality from SCD due to VT/VF (Al-Khatib et al., 2018), yet implantable cardioverter defibrillator (ICD) shocks are painful, and are associated with significant morbidity and poor quality of life (Al-Khatib et al., 2018). Therefore, preventive strategies to decrease ICD shocks are imperative in the management of these patients (Al-Khatib et al., 2018) and highlight the need for alternative treatment modalities.

## Methods

### Patient Enrollment and Study Protocol

This was a prospective pilot study (NCT03549468). Patients with ICM (left ventricular ejection fraction <35%) and HF who had an implantable device with an atrial lead (dual chamber ICD or cardiac resynchronization therapy) were enrolled in the study. In addition, patients were required to be in sinus rhythm at the time of the study visit. Patients were excluded if: they had recent (<6 months) stroke or myocardial infarction, had persistent AF, had recurrent vaso-vagal syncopal episodes, had undergone unilateral or bilateral vagotomy, were in pregnancy or breast feeding, had history of uncontrolled diabetes or hypertension, or were hypotensive due to autonomic dysfunction. All patients provided informed consent prior to enrollment in the study which was approved by the Institutional Review Board of the University of Oklahoma Health Sciences Center.

**FIGURE 1:**
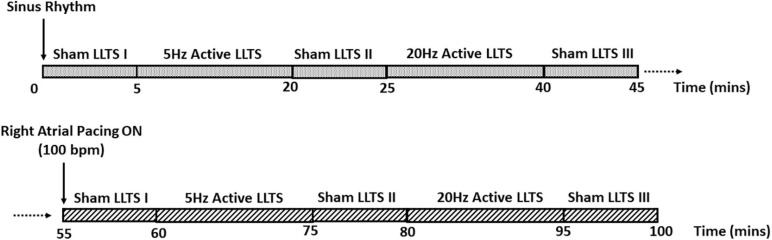
Schematic of the study protocol. Each patient enrolled in the study first received 15 min of active LLTS at 5 Hz and 20 Hz, during sinus rhythm. 5 minutes of sham LLTS was performed before and after each active LLTS acquisition. After a 10-minute wash-out period, the 45-minute stimulation protocol was repeated with concomitant atrial pacing at 100 bpm.

LLTS was delivered as previously described (Stavrakis et al., 2015, 2020b) through a transcutaneous electrical nerve stimulation (TENS) device (Parasym device, Parasym Health, Inc., London, United Kingdom), at a pulse width of 200 μs and a pulse frequency of 5 Hz or 20 Hz. The stimulation amplitude was individually titrated to 1 mA below the discomfort threshold based on individual patient’s perception. Active LLTS was accomplished by attaching an ear clip to the tragus, known to be innervated by the auricular branch of the vagus nerve (Peuker and Filler, 2002). For sham stimulation, the electrode was attached to the tragus, but no stimulation was delivered. A schematic of the study protocol is presented in Figure 1.

Briefly, each patient received 15 min of active LLTS at 5 Hz and 20 Hz; 5 min of sham stimulation was performed before and after each active LLTS period; 45 min of continuous, high resolution ECG (12 lead ECG recorder, IX-ECG12, iWorx, 62 Littleworth Road, Dover, NH 03820, United States, sampling rate 10,000 Hz) was acquired for TWA analysis during sinus rhythm, followed by a 10 min wash-out period. Next, the same stimulation protocol was repeated, with concomitant atrial pacing at 100 bpm, to elicit TWA, as previously described (Kaufman et al., 2000; Armoundas et al., 2013; Merchant et al., 2020). Patients with significant premature ventricular contractions, AV nodal dysfunction, ventricular pacing or significant stimulation artifacts were excluded from the TWA analysis. Hence, TWA results were available for 19 patients during sinus rhythm and 17 patients during atrial pacing. Furthermore, atrial pacing was performed at a rate as close to 100 bpm as possible (for more details, please see the Supplementary Material), without inducing Wenckebach AV block or other adverse effects.

### Estimation of Repolarization Alternans Burden

We have previously demonstrated the ability of microvolt TWA to predict short- (Weiss et al., 2011; Merchant and Armoundas, 2012; Merchant et al., 2013b, 2020) and long-term (Merchant et al., 2012, 2015; Sohn et al., 2019) susceptibility to ventricular tachy-arrhythmias and sudden cardiac death, using an algorithm based on the spectral method (Smith et al., 1988; Merchant and Armoundas, 2012; Merchant et al., 2012, 2013b, 2015; Sohn et al., 2019). Briefly, the alternans voltage was used as a direct measure of the presence of alternans and calculated based on the amplitude of the power spectrum at the alternans frequency (0.5 cycles/beat) (Armoundas et al., 2013; Sayadi et al., 2013; Merchant et al., 2014, 2020). The alternans ratio, *K*_score_, was used as a statistical measure of alternans calculated as the alternans voltage relative to the background noise level (Armoundas et al., 2013; Sayadi et al., 2013; Merchant et al., 2014, 2020). TWA voltage and *K*_score_ was estimated using the spectral method for each moving window of 128-beat data sequence, advanced one beat at a time, using a 512-point power spectrum to improve frequency-domain resolution.

TWA burden was calculated for each of the 12 leads, for each intervention, based on estimates of the alternans voltage and *K*_score_, for each patient, as follows:

TWA burden = (positive TWA sequences/total number of sequences) × 100%

where, a positive TWA sequence was defined as any 128 beat sequence with *K*_score_ > 3, alternans voltage >1.0 uV and goodbeat% >80. Goodbeat percentage was calculated for all sequences as a moving average of the number of good beats (correlation with average QRS template >0.90 and the difference between current R-to-R waveform interval and the median RR interval from the preceding 7 beats <10%).

### Statistical Analysis

Analysis was performed on a lead-by-lead basis and TWA burden for each lead was calculated across all patients during the five interventions: (1) Sham I, (2) 5 Hz Active LLTS, (3) Sham II, (4) 20 Hz Active LLTS, and (5) Sham III. While data for all 12 leads was individually analyzed, data were aggregated across the precordial and limb leads, to increase the power to detect differences in TWA burden. Significant alternans levels were observed primarily in the precordial leads, therefore, summary results of TWA burden distributions are presented only for the precordial leads, with data grouped across leads V1–V6.

**FIGURE 2:**
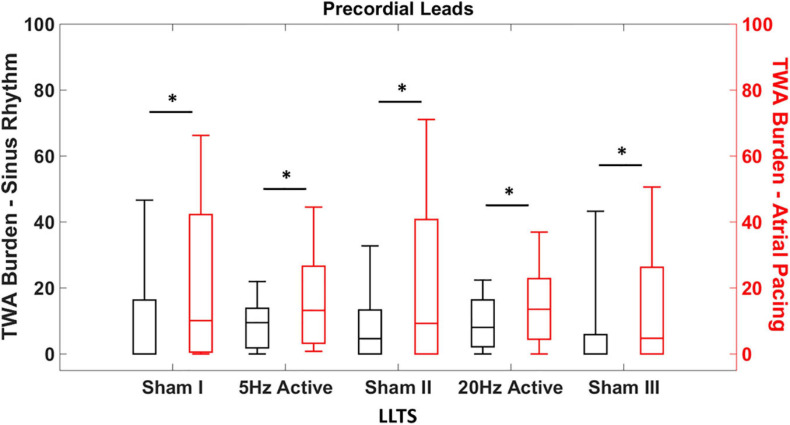
Heart rate dependent effects of tragus stimulation on T-wave alternans (TWA). Quantification of the TWA burden during sinus rhythm and atrial pacing (100 bpm), with sham and active LLTS. TWA burden is significantly elevated in the precordial leads irrespective of LLTS during atrial pacing, compared to sinus rhythm. “*” denotes statistical significance of *p* < 0.05 using Kruskal-Wallis test.

**FIGURE 3:**
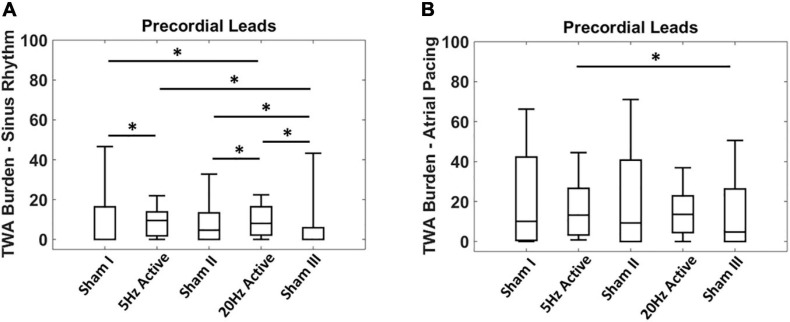
Acute effects of low level tragus stimulation (LLTS) on T-wave alternans (TWA) burden. **(A)** TWA burden is significantly elevated with active LLTS at 5 Hz and 20 Hz, compared to sham LLTS during sinus rhythm. **(B)** Higher baseline TWA burden is observed during 100 bpm atrial pacing with no enhancement in TWA burden observed with active LLTS. “*” denotes statistical significance of *p* < 0.05 using Wilcoxon rank sum test.

Given the non-normal distribution of the TWA burden, the non-parametric Kruskal-Wallis test was used to compare the TWA burden during sinus rhythm with the concomitant atrial pacing at 100 bpm (Figure 2). Inter-interventional comparison of TWA burden was performed using the Wilcoxon rank sum test (Figure 3). All analysis was performed using MATLAB (MathWorks Inc., Natick, MA, United States) and values of *p* < 0.05 were considered statistically significant.

## Results

### Patient Characteristics

The baseline clinical characteristics of the patients enrolled in this study are summarized in Table 1. Briefly, the patients were elderly (mean age 66.9 ± 8.2 years), predominantly male (77%), with prevalent hypertension (77%), hyperlipidemia (82%), and diabetes (38%), with mean LV ejection fraction 30.4 ± 9.3%. None of the patients reported angina.

**TABLE 1:** Baseline characteristics of the study population (*n* = 26).

Age (years)	66.9 ± 8.2
Female sex, n (%)	6 (23)
White race, n (%)	23 (89)
**Type of device**	
Implantable cardioverter defibrillator, n (%)	19 (73)
Cardiac resynchronization therapy, n (%)	7 (27)
Body mass index (Kg/m^2^)	29.9 ± 5.1
Left ventricular ejection fraction (%)	30.4 ± 9.3
Coronary artery bypass graft surgery, n (%)	13 (50)
Diabetes, n (%)	10 (38)
Hyperlipidemia, n (%)	22 (85)
Hypertension, n (%)	20 (77)
Chronic kidney disease, n (%)	8 (31)
**New York Heart Association class, n (%)**	
I	8 (31)
II	14 (54)
III	4 (15)
**Medications**	
Beta blockers, n (%)	24 (92)
Angiotensin converting enzyme inhibitors/Angiotensin receptor blockers, n (%)	24 (92)
Spironolactone, n (%)	9 (35)
Statins, n (%)	22 (85)
Hgb (g/dl)	13.7 ± 2.6
Creatinine (mg/dL)	1.4 ± 0.9
Sodium (mEq/L)	139.9 ± 2.8
Potassium (mEq/L)	4.2 ± 0.4
Brain natriuretic peptide (pg/ml)	324.6 ± 465.7

*Continuous data are presented as mean ± standard deviation.*

### Comparison of the Effects of Acute Low Level Tragus Stimulation During Sinus Rhythm and Atrial Pacing

We first determined whether the effect of LLTS on TWA was HR dependent. The individual heart rates for each patient during sinus rhythm and atrial pacing, for all interventions are presented in Supplementary Table 1. Expectedly, the heart rates during sinus rhythm were significantly lower than during atrial pacing for each intervention (Supplementary Table 1). Figure 2 demonstrates summary results of TWA burden across all patients during sinus rhythm (black) and during atrial pacing at 100 bpm (red). Data are presented as median (horizontal solid line), 75–25% percentiles (box) and 90–10% percentiles (error bars). Significantly higher levels of TWA burden were observed during atrial pacing compared to sinus rhythm, during all five interventions, demonstrating the dependency of alternans onset on the underlying HR and subsequently the effect of LLTS on modulation of TWA.

### Effect of Acute Low Level Tragus Stimulation on T-Wave Alternans Burden

Next, we evaluated the effect of the LLTS frequency, on TWA burden. Figure 3A shows summary results of acute LLTS at 5 Hz and 20 Hz, on TWA in the precordial leads, during sinus rhythm. Data are presented as median (horizontal solid line), 75–25% percentiles (box) and 90–10% percentiles (error bars). Active LLTS at both 5 Hz and 20 Hz led to a significant rise in TWA burden, compared to sham LLTS. This effect was no longer present during atrial pacing (Figure 3B), when either 5 Hz or 20 Hz active LLTS had no significant effect of TWA burden. Effect of LLTS on TWA burden of each individual lead during both sinus rhythm and atrial pacing is presented in Supplementary Table 2.

## Discussion

Microvolt TWA is a surrogate marker for susceptibility to VT/VF, leading to SCD (Merchant et al., 2012, 2013a, 2015; Kulkarni et al., 2019). Favorable modulation of VT/VF susceptibility by LLTS in a population at high risk for VT/VF such as patients with ICM may be a surrogate for potential benefit in a larger number of patients at risk for VT/VF. A comprehensive clinical evaluation of the effects of LLTS on cardiac arrhythmogenesis can aid treatment of patients with chronic underlying - cardiovascular diseases (De Ferrari et al., 2011). The major findings of our preliminary study are, *first*, acute LLTS modulates TWA burden in patients with ICM and HF; *second*, effect of LLTS on TWA is modulated by the HR; and *third*, increased HR leads to an increase in TWA burden in patients with ICM and HF, possibly masking the effects of acute LLTS.

While the results of our study seem counterintuitive with acute LLTS eliciting a rise in TWA burden, the current results are in line with a recent study investigating the effect of LLTS on atrial alternans and AF burden in patients with paroxysmal AF (Kulkarni et al., 2021). A biphasic response to LLTS was observed in this proof-of-concept study wherein acute LLTS led to a rise in atrial alternans level, but chronic LLTS over 6 months, significantly reduced both alternans and AF burden. Given the disparate effects of acute and chronic parasympathetic stimulation, it is possible that chronic LLTS could favorably modulate TWA burden in patients with HF and demonstrate the expected anti-arrhythmic results. Furthermore, VNS has been shown to affect both atrial and ventricular electrophysiology (Schwartz and Zipes, 2000; Ng et al., 2007; Stavrakis et al., 2020a), and modulate both P-wave and T-wave alternans in various patient populations.

It is known that the effect of VNS depends on the stimulation parameters (Ardell et al., 2017) which may partly explain the inconsistent results of clinical trials aimed at investigating the utility of VNS in treating cardiovascular diseases (Premchand et al., 2014; Zannad et al., 2015; Gold et al., 2016). Response to VNS can be patient-specific, requiring the use of optimal stimulation parameters tailored to elicit the ideal response from each patient (Ardell et al., 2017). Since standard stimulation frequencies are yet to be established, in our study we used the LLTS frequencies (5 Hz and 20 Hz) used in clinical trials investigating the effect of VNS in HF, covering a wide physiological range, in an attempt to elucidate a potential frequency dependent effect of LLTS on TWA modulation. However, we observed that both 5 Hz and 20 Hz acute LLTS led to a similar rise in TWA burden during sinus rhythm.

TWA burden was observed to be higher during atrial pacing at faster HR, which is consistent with previous findings and known restitution mediated mechanisms of TWA onset (Ng et al., 2007; Merchant et al., 2012, 2013a; Kulkarni et al., 2019). Hence, the lack of significant effects of LLTS on TWA burden during atrial pacing compared to sinus rhythm could be attributed to higher baseline levels of alternans. This indicates that the effects of LLTS on TWA is HR dependent and consequently the parasympathetic stimulation parameters would need to be chosen taking into account the underlying patient-specific HR dynamics.

### Clinical Implications

The distinct and significant modulation of TWA by acute LLTS, in conjunction with the bimodal effect of LLTS on atrial alternans (Kulkarni et al., 2021) demonstrates a possible pathway for designing an anti-arrhythmic treatment strategy for patients with ICM and HF based on parasympathetic stimulation. Given that LLTS is associated with minimal risk, based on our prior experience (Stavrakis et al., 2020b), such a treatment may have important clinical implications. Moreover, short-term (1 h daily) LLTS application has been shown to achieve long-lasting antiarrhythmic effects (Stavrakis et al., 2020b), consistent with the notion that the LLTS effects exhibit memory (Stavrakis et al., 2020a). Nonetheless, the minimal duration of LLTS that is required to affect clinical outcomes remains to be determined. This study provides insights for a more comprehensive trial to evaluate the effects of chronic LLTS in patients susceptible to ventricular tachy-arrhythmias, as well as to investigate the potential of patient-specific stimulation strategies in evoking the optimal response to LLTS.

### Limitations

This study has several limitations. First, this was a pilot study in a small patient cohort. Second, we chose to evaluate continuous LLTS at only two frequencies, 5 Hz and 20 Hz, with a fixed pulse width (200 μs), for a duration of 15 min. Third, the ideal wash out period for LLTS is not known. We chose to limit the sham periods to 5 min, in order to keep the duration of the experimental protocol within reasonable time limits. Fourth, we did not evaluate the effects of chronic LLTS on TWA; given that LLTS could have a biphasic response, further validation is required in a larger cohort to evaluate its chronic effects. Finally, an investigation of the effects of LLTS on heart rate variability dynamics could help provide a physiological assessment of the autonomic modulation of cardiac arrhythmias.

## Conclusion

Acute LLTS results in increased TWA burden during sinus rhythm in patients with ICM and HF, and this effect is HR dependent, with atrial pacing at 100 bpm resulting in elevated TWA burden compared to sinus rhythm.

## Data Availability Statement

The raw data supporting the conclusion of this article will be made available by the authors, without undue reservation.

## Ethics Statement

The studies involving human participants were reviewed and approved by Institutional Review Board of The University of Oklahoma Health Sciences Center. The patients/participants provided their written informed consent to participate in this study.

## Author Contributions

KK participated in the development of the algorithms, data analysis, and writing the manuscript. SS participated in the conception of the study, the data collection, and writing the manuscript. KE participated in the data collection and writing the manuscript. JS and KP participated in the writing of the manuscript. AA participated in the conception and funding of the study, the development of the algorithms, data analysis, and writing the manuscript. All authors wrote the manuscript, contributed to the article, and approved the submitted version.

## Conflict of Interest

The authors declare that the research was conducted in the absence of any commercial or financial relationships that could be construed as a potential conflict of interest.

## Publisher’s Note

All claims expressed in this article are solely those of the authors and do not necessarily represent those of their affiliated organizations, or those of the publisher, the editors and the reviewers. Any product that may be evaluated in this article, or claim that may be made by its manufacturer, is not guaranteed or endorsed by the publisher.

## References

[B1] Al-Khatib S. M. Stevenson W. G. Ackerman M. J. Bryant W. J. Callans D. J. Curtis A. B. (2018). AHA/ACC/HRS guideline for management of patients with ventricular arrhythmias and the prevention of sudden cardiac death: a report of the American college of cardiology/American heart association task force on clinical practice guidelines and the heart rhythm society. *J. Am. Coll. Cardiol.* 72 e91–e220. 29097296 10.1016/j.jacc.2017.10.054

[B2] Ardell J. L. Nier H. Hammer M. Southerland E. M. Ardell C. L. Beaumont E. (2017). Defining the neural fulcrum for chronic vagus nerve stimulation: implications for integrated cardiac control. *J. Physiol.* 595 6887–6903. 10.1113/jp274678 28862330 PMC5685838

[B3] Armoundas A. A. Weiss E. H. Sayadi O. Laferriere S. Sajja N. Mela T. (2013). A novel pacing method to suppress repolarization alternans in vivo: implications for arrhythmia prevention. *Heart Rhythm.* 10 564–572. 10.1016/j.hrthm.2012.12.026 23274372

[B4] Billman G. E. (2006). A comprehensive review and analysis of 25 years of data from an in vivo canine model of sudden cardiac death: implications for future anti-arrhythmic drug development. *Pharmacol. Ther.* 111 808–835. 10.1016/j.pharmthera.2006.01.002 16483666

[B5] De Ferrari G. M. Crijns H. J. Borggrefe M. Milasinovic G. Smid J. Zabel M. (2011). Chronic vagus nerve stimulation: a new and promising therapeutic approach for chronic heart failure. *Eur. Heart J.* 32 847–855. 10.1093/eurheartj/ehq391 21030409

[B6] Gold M. R. Van Veldhuisen D. J. Hauptman P. J. Borggrefe M. Kubo S. H. Lieberman R. A. (2016). Vagus nerve stimulation for the treatment of heart failure: the INOVATE-HF trial. *J. Am. Coll. Cardiol.* 68 149–158. 27058909 10.1016/j.jacc.2016.03.525

[B7] Kaufman E. S. Mackall J. A. Julka B. Drabek C. Rosenbaum D. S. (2000). Influence of heart rate and sympathetic stimulation on arrhythmogenic T wave alternans. *Am. J. Physiol. Heart Circulatory Physiol.* 279 H1248–H1255. 10.1152/ajpheart.2000.279.3.H1248 10993791

[B8] Klingenheben T. Gronefeld G. Li Y. G. Hohnloser S. H. (2001). Effect of metoprolol and d,l-sotalol on microvolt-level T-wave alternans. results of a prospective, double-blind, randomized study. *J. Am. Coll. Cardiol.* 38 2013–2019. 10.1016/s0735-1097(01)01661-8 11738309

[B9] Komiya N. Seto S. Nakao K. Yano K. (2005). The influence of beta-adrenergic agonists and antagonists on T-wave alternans in patients with and without ventricular tachyarrhythmia. *Pacing Clin. Electrophysiol.* 28 680–684. 10.1111/j.1540-8159.2005.00146.x 16008804

[B10] Kulkarni K. Merchant F. M. Kassab M. B. Sana F. Moazzami K. Sayadi O. (2019). Cardiac alternans: mechanisms and clinical utility in arrhythmia prevention. *J. Am. Heart Assoc.* 8:e013750. 31617437 10.1161/JAHA.119.013750 PMC6898836

[B11] Kulkarni K. Singh J. P. Parks K. A. Katritsis D. G. Stavrakis S. Armoundas A. A. (2021). Low-level tragus stimulation modulates atrial alternans and fibrillation burden in patients with paroxysmal atrial fibrillation. *J. Am. Heart Assoc.* 10:e020865. 34075778 10.1161/JAHA.120.020865 PMC8477868

[B12] Lampert R. Shusterman V. Burg M. M. Lee F. A. Earley C. Goldberg A. (2005). Effects of psychologic stress on repolarization and relationship to autonomic and hemodynamic factors. *J. Cardiovasc. Electrophysiol.* 16 372–377. 10.1046/j.1540-8167.2005.40580.x 15828878

[B13] Libbus I. Nearing B. D. Amurthur B. KenKnight B. H. Verrier R. L. (2016). Autonomic regulation therapy suppresses quantitative T-wave alternans and improves baroreflex sensitivity in patients with heart failure enrolled in the ANTHEM-HF study. *Heart Rhythm* 13 721–728. 10.1016/j.hrthm.2015.11.030 26601770

[B14] Merchant F. M. Armoundas A. A. (2012). Role of substrate and triggers in the genesis of cardiac alternans, from the myocyte to the whole heart: implications for therapy. *Circulation* 125 539–549. 10.1161/circulationaha.111.033563 22271847 PMC3281422

[B15] Merchant F. M. Ikeda T. Pedretti R. F. Salerno-Uriarte J. A. Chow T. Chan P. S. (2012). Clinical utility of microvolt T-wave alternans testing in identifying patients at high or low risk of sudden cardiac death. *Heart Rhythm* 9 1256–1264 e2. 22406384 10.1016/j.hrthm.2012.03.014 PMC3411866

[B16] Merchant F. M. Salerno-Uriarte J. A. Caravati F. Falcone S. Molon G. Marangoni D. (2015). Prospective use of microvolt t-wave alternans testing to guide primary prevention implantable cardioverter defibrillator therapy. *Circ. J.* 79 1912–1919. 10.1253/circj.cj-15-0253 26073692

[B17] Merchant F. M. Sayadi O. Moazzami K. Puppala D. Armoundas A. A. (2013a). T-wave alternans as an arrhythmic risk stratifier: state of the art. *Curr. Cardiol. Rep.* 15:398. 23881581 10.1007/s11886-013-0398-7 PMC3763749

[B18] Merchant F. M. Sayadi O. Puppala D. Moazzami K. Heller V. Armoundas A. A. (2014). A translational approach to probe the proarrhythmic potential of cardiac alternans: a reversible overture to arrhythmogenesis? *Am. J. Physiol. Heart Circ. Physiol.* 306 H465–H474. 24322612 10.1152/ajpheart.00639.2013 PMC3920241

[B19] Merchant F. M. Sayadi O. Sohn K. Weiss E. H. Puppala D. Doddamani R. (2020). Real-time closed-loop suppression of repolarization alternans reduces arrhythmia susceptibility in vivo. *Circ. Arrhythm. Electrophysiol.* 13:e008186. 32434448 10.1161/CIRCEP.119.008186 PMC7334752

[B20] Merchant F. M. Zheng H. Bigger T. Steinman R. Ikeda T. Pedretti R. F. (2013b). A combined anatomic and electrophysiologic substrate based approach for sudden cardiac death risk stratification. *Am. Heart J.* 166 744–752. 10.1016/j.ahj.2013.06.023 24093856 PMC4188429

[B21] Myerburg R. J. Junttila M. J. (2012). Sudden cardiac death caused by coronary heart disease. *Circulation* 125 1043–1052. 10.1161/circulationaha.111.023846 22371442

[B22] Nearing B. D. Wellenius G. A. Mittleman M. A. Josephson M. E. Burger A. J. Verrier R. L. (2012). Crescendo in depolarization and repolarization heterogeneity heralds development of ventricular tachycardia in hospitalized patients with decompensated heart failure. *Circ. Arrhythm. Electrophysiol.* 5 84–90. 10.1161/circep.111.965434 22157521 PMC3296063

[B23] Ng G. A. Brack K. E. Patel V. H. Coote J. H. (2007). Autonomic modulation of electrical restitution, alternans and ventricular fibrillation initiation in the isolated heart. *Cardiovasc. Res.* 73 750–760. 10.1016/j.cardiores.2006.12.001 17217937

[B24] MERIT-HF (1999). Effect of metoprolol CR/XL in chronic heart failure: metoprolol CR/XL randomised intervention trial in congestive heart failure (MERIT-HF). *Lancet* 353 2001–2007. 10.1016/s0140-6736(99)04440-2 10376614

[B25] Olshansky B. Sabbah H. N. Hauptman P. J. Colucci W. S. (2008). Parasympathetic nervous system and heart failure: pathophysiology and potential implications for therapy. *Circulation* 118 863–871. 10.1161/circulationaha.107.760405 18711023

[B26] Peuker E. T. Filler T. J. (2002). The nerve supply of the human auricle. *Clin. Anat.* 15 35–37. 10.1002/ca.1089 11835542

[B27] Poole-Wilson P. A. Swedberg K. Cleland J. G. Di Lenarda A. Hanrath P. Komajda M. (2003). Comparison of carvedilol and metoprolol on clinical outcomes in patients with chronic heart failure in the Carvedilol Or Metoprolol European Trial (COMET): randomised controlled trial. *Lancet* 362 7–13. 10.1016/s0140-6736(03)13800-7 12853193

[B28] Premchand R. K. Sharma K. Mittal S. Monteiro R. Dixit S. Libbus I. (2014). Autonomic regulation therapy via left or right cervical vagus nerve stimulation in patients with chronic heart failure: results of the ANTHEM-HF trial. *J. Card. Fail* 20 808–816. 10.1016/j.cardfail.2014.08.009 25187002

[B29] Rashba E. J. Cooklin M. MacMurdy K. Kavesh N. Kirk M. Sarang S. (2002). Effects of selective autonomic blockade on T-wave alternans in humans. *Circulation* 105 837–842. 10.1161/hc0702.104127 11854124

[B30] Rubart M. Zipes D. P. (2005). Mechanisms of sudden cardiac death. *J. Clin. Invest.* 115 2305–2315. 16138184 10.1172/JCI26381 PMC1193893

[B31] Sayadi O. Merchant F. M. Puppala D. Mela T. Singh J. P. Heist E. K. (2013). A novel method for determining the phase of T-wave alternans: diagnostic and therapeutic implications. *Circ. Arrhythm Electrophysiol.* 6 818–826. 10.1161/circep.113.000114 23884196 PMC3845209

[B32] Schwartz P. J. Zipes D. P. (2000). “Autonomic modulation of cardiac arrhythmias,” in *Cardiac Electophysiology: from Cell to Bedside*, 3rd Edn, eds Zipes D. P. Jalife J. (Amsterdam: Elsevier).

[B33] Shusterman V. Goldberg A. London B. (2006). Upsurge in T-wave alternans and nonalternating repolarization instability precedes spontaneous initiation of ventricular tachyarrhythmias in humans. *Circulation* 113 2880–2887. 10.1161/circulationaha.105.607895 16785339

[B34] Smith J. M. Clancy E. A. Valeri C. R. Ruskin J. N. Cohen R. J. (1988). Electrical alternans and cardiac electrical instability. *Circulation* 77 110–121. 10.1161/01.cir.77.1.110 3335062

[B35] Sohn K. Dalvin S. P. Merchant F. M. Kulkarni K. Sana F. Abohashem S. (2019). Utility of a smartphone based system (cvrphone) to predict short-term arrhythmia susceptibility. *Sci. Rep.* 9:14497. 31601824 10.1038/s41598-019-50487-4 PMC6787075

[B36] Stavrakis S. Humphrey M. B. Scherlag B. J. Hu Y. Jackman W. M. Nakagawa H. (2015). Low-level transcutaneous electrical vagus nerve stimulation suppresses atrial fibrillation. *J. Am. Coll. Cardiol.* 65 867–875. 10.1016/j.jacc.2014.12.026 25744003 PMC4352201

[B37] Stavrakis S. Kulkarni K. Singh J. P. Katritsis D. G. Armoundas A. A. (2020a). Autonomic modulation of cardiac arrhythmias: methods to assess treatment and outcomes. *JACC Clin. Electrophysiol.* 6 467–483. 32439031 10.1016/j.jacep.2020.02.014 PMC7370838

[B38] Stavrakis S. Stoner J. A. Humphrey M. B. Morris L. Filiberti A. Reynolds J. C. (2020b). TREAT AF (transcutaneous electrical vagus nerve stimulation to suppress atrial fibrillation): a randomized clinical trial. *JACC Clin. Electrophysiol.* 6 282–291. 10.1016/j.jacep.2019.11.008 32192678 PMC7100921

[B39] Verrier R. L. Antzelevitch C. (2004). Autonomic aspects of arrhythmogenesis: the enduring and the new. *Curr. Opin. Cardiol.* 19 2–11. 10.1097/00001573-200401000-00003 14688627 PMC1513619

[B40] Weiss E. H. Merchant F. M. d’Avila A. Foley L. Reddy V. Y. Singh J. P. (2011). A novel lead configuration for optimal spatio-temporal detection of intracardiac repolarization alternans. *Circ. Arrhythm. Electrophysiol.* 4 407–417. 10.1161/circep.109.934208 21430127 PMC3148443

[B41] Zannad F. De Ferrari G. M. Tuinenburg A. E. Wright D. Brugada J. Butter C. (2015). Chronic vagal stimulation for the treatment of low ejection fraction heart failure: results of the NEural Cardiac TherApy foR Heart Failure (NECTAR-HF) randomized controlled trial. *Eur. Heart J.* 36 425–433. 10.1093/eurheartj/ehu345 25176942 PMC4328197

