# Supplementary material for: Microvolt T-Wave Alternans Is Modulated by Acute Low-Level Tragus Stimulation in Patients With Ischemic Cardiomyopathy and Heart Failure
Source: Front Physiol. 2021 Jul 23;12:707724. doi: 10.3389/fphys.2021.707724 (PMC8343129; doi:10.3389/fphys.2021.707724)
Supplement: Supplementary file 1 [file Data_Sheet_1.pdf]

Online Supplement

Microvolt T-wave Alternans is Modulated by Acute Low-Level Tragus Stimulation in Patients with Ischemic Cardiomyopathy and Heart Failure

Kanchan Kulkarni, PhD, Stavros Stavrakis, MD, PhD, Khaled Elkholey, MD, Jagmeet P. Singh, MD, PhD, Kimberly A. Parks, DO, Antonis A. Armoundas, PhD

RESULTS

Effect on heart rate

Table 1 presents the heart rate during sinus rhythm and with concomitant atrial pacing for each patient during each intervention. Mean, standard deviation and standard error in heart rates for each intervention are also presented. Heart rates during sinus rhythm are significantly lower than during atrial pacing for each intervention.

Effect on T-wave alternans burden

Table 2 presents the median T-wave alternans (TWA) burden observed in each lead across all patients during sinus rhythm and with concomitant atrial pacing. Mean, standard deviation and standard error in median TWA burden for each intervention are also presented. Acute low-level tragus stimulation (LLTS) increases TWA burden compared to sham during sinus rhythm. This effect is diminished with atrial pacing at faster heart rates.

Online Supplement

Table 1. Heart rates during sinus rhythm and atrial pacing (n=19). Mean heart rate for each patient during each intervention. '-' denotes no response to atrial pacing. Patients with significant premature ventricular contractions, AV nodal dysfunction, ventricular pacing or significant stimulation artifacts were excluded from the analysis. * denotes statistical significance of $p < 0.05$ between Sham I interventions, during sinus rhythm and atrial pacing, # denotes statistical significance of $p < 0.05$ between 5Hz LLTS interventions, during sinus rhythm and atrial pacing, $ denotes statistical significance of $p < 0.05$ between Sham II interventions, during sinus rhythm and atrial pacing, ^ denotes statistical significance of $p < 0.05$ between 20Hz LLTS interventions, during sinus rhythm and atrial pacing, and & denotes statistical significance of $p < 0.05$ between Sham III interventions, during sinus rhythm and atrial pacing (1-way ANOVA).

	Sinus Rhythm (bpm)					Atrial Pacing (bpm)				
Patient	Sham I	5 Hz LLTS	Sham II	20 Hz LLTS	Sham III	Sham I	5 Hz LLTS	Sham II	20 Hz LLTS	Sham III
3	67	66	67	66	65	-	-	-	-	-
5	66	68	67	67	66	100	100	100	100	100
6	60	60	61	62	60	100	100	100	100	100
7	70	71	71	70	69	-	90	90	90	90
9	63	62	61	62	62	100	100	100	100	100
10	54	53	53	53	52	90	90	90	90	90
12	59	58	57	56	58	100	100	100	100	100
13	75	76	76	77	77	100	100	100	100	100
14	60	60	60	60	60	100	100	100	100	100
17	53	51	52	51	50	100	100	100	100	100
18	78	78	79	77	78	100	100	100	100	99
19	50	50	50	50	50	90	90	90	90	90
20	57	57	57	57	57	100	100	100	100	100
21	65	64	62	64	63	100	100	100	100	100
22	77	77	78	78	79	79	80	81	81	82
23	50	50	50	50	50	90	90	90	90	90
24	50	50	50	50	50	-	-	-	-	-
25	56	57	56	56	57	100	100	100	100	100
26	61	68	59	62	61	100	100	101	100	100
Mean	61.63*	61.89#	61.37$	61.47^	61.26&	96.81*	96.47#	96.59$	96.53^	96.53&
Stdev.	8.88	9.3	9.36	9.26	9.39	6.21	6.06	5.94	5.9	5.7
Error	2.04	2.13	2.15	2.12	2.15	1.55	1.47	1.44	1.43	1.38

Table 2. T-wave alternans (TWA) burden during sinus rhythm and atrial pacing (n=19). Median TWA burden (%) observed in each lead during each intervention. *, #, $, ^ and & denote statistical significance of $p < 0.05$ between respective pairs of interventions, during sinus rhythm and atrial pacing (1-way ANOVA, Tukey post hoc test).

	Sinus Rhythm TWA Burden (%)					Atrial Pacing TWA Burden (%)				
Lead	Sham I	5 Hz LLTS	Sham II	20 Hz LLTS	Sham III	Sham I	5 Hz LLTS	Sham II	20 Hz LLTS	Sham III
Lead I	10.77	3.72	0	2.03	0	7.41	9.39	6.95	18.82	3.29
Lead II	0.51	6.9	2.3	3.32	0	14.62	10.65	18.24	13.38	0.5
Lead III	10.48	3.14*	0	4.15	0*	17.74	14.34	4.71	12.8	11.87
aVR	0	7.33*	0*	4.4	1.32	11.04	8.29	8.04	11.64	0.85
aVL	2.22	6.72	5.67	4.33	0	$4.86^{\$}$	11.18	7.6	$21.86^{\$}$	3.62
aVF	0	5.36*	$0*^{\#}$	$7.56^{\#}$	0	$8.37^{\$}$	8.1	8.77	$19.68^{\$}$	12.84
V1	1.11	11.65	3.97	5.25	0	1.74	8.51	4.73	6.95	0
V2	0	9.53*	$0.76^{\#}$	$14.08^{\#\$}$	$0*^{\$}$	20.54	17.9	7.1	7.73	1.83
V3	0.28	9.73	9.51	$7.19^{\$}$	$0.29^{\$}$	10.42	14.11	15.9	17	13.74
V4	2.19	2.87	0	10.42	0	4.38	15.64	9.79	25.2	8.72
V5	0	9.5	10.54	6.43	1.54	8.78	14.36	3.8	12.45	2.71
V6	2.92	7.35*	4.36	$4.87^{\$}$	$0*^{\$}$	18.13	10.7	20.61	15.88	8.75

Mean	$2.54*^{\$}$	$6.98*^{\wedge\&}$	$3.09^{\wedge}$	$6.17^{\$\#}$	$0.26^{\&\#}$	10.67	11.93*	9.69	$15.28^{\$}$	$5.73*^{\$}$
Stdev.	3.91	2.82	3.81	3.33	0.55	5.97	3.24	5.54	5.51	5.13
Error	1.13	0.81	1.1	0.96	0.16	1.72	0.94	1.6	1.59	1.48
